# Cervical esophageal perforation caused by the use of bougie during laparoscopic sleeve gastrectomy: a case report and review of the literature

**DOI:** 10.1186/s12893-020-0679-1

**Published:** 2020-01-10

**Authors:** Andrea Lovece, Ioannis Rouvelas, Masaru Hayami, Mats Lindblad, Andrianos Tsekrekos

**Affiliations:** 1 Division of General Surgery, IRCCS Policlinico San Donato, San Donato Milanese, Italy; 2 Division of Surgery, Department of Clinical Science, Intervention and Technology (CLINTEC), Karolinska Institutet, Stockholm, Sweden; 3 Department of Upper Abdominal Surgery, Karolinska University Hospital, K42, 14186 Stockholm, Sweden

**Keywords:** Esophageal perforation, Laparoscopic sleeve gastrectomy, Bougie, Case report

## Abstract

**Background:**

Obesity is considered a chronic disease with an increasing prevalence worldwide during the last decades. Laparoscopic sleeve gastrectomy is the most commonly performed bariatric procedure, due to its relative safety and long-term efficacy. The use of bougie to ensure correct size of the gastric tube is part of the standard operation, usually placed by the anesthesiologist and with a very low rate of complications. We report the first case, to our knowledge, of a cervical esophageal perforation caused by the use of bougie during laparoscopic sleeve gastrectomy.

**Case presentation:**

The complication occurred in a previously healthy 42-year old female patient who underwent laparoscopic sleeve gastrectomy for class 1 obesity (BMI 31 kg/m^2^) and was diagnosed the first post-operative day. She was subsequently treated with an emergency thoracoscopy and evacuation of a mediastinal fluid collection, with additional neck incision for primary closure of the esophageal defect which was reinforced with a sternocleidomastoid muscle flap. The post-operative course was uneventful.

**Conclusions:**

We made a literature review to better understand the options considering the diagnosis and treatment in case of very proximal iatrogenic esophageal perforations. The risks related to the use of bougie during surgery should not be underestimated, and its insertion must be done with extreme caution. Esophageal perforation is still a challenging, life threatening complication where prompt diagnosis and adequate treatment are essential.

## Background

Obesity is a chronic disease that is constantly increasing in prevalence around the world and is now considered to be a worldwide epidemic. Recent studies reported that in 2015, approximately 108 million children and 604 million adults globally were obese [1].

During the last years, laparoscopic sleeve gastrectomy (LSG) has become the most commonly performed bariatric procedure in the world [2]. This procedure is relatively safe and effective, providing a high rate of weight loss and lasting long-term results [3]. Regarding the surgical technique, there is consensus on the use of a bougie placed along the lesser curvature in order to calibrate the width of the remaining stomach during the resection. Despite that this is a very common practice, esophageal perforations following the use of bougie are extremely rare, with only a few cases reported in the literature. To our knowledge, we report the first case of a cervical esophageal perforation during LSG, caused by the use of bougie and diagnosed on the first post-operative day. We also discuss the possible diagnostic and treatment options, in order to conclude which is the best approach when a similar life-threatening complication occurs.

## Case presentation

**Fig. 1:**
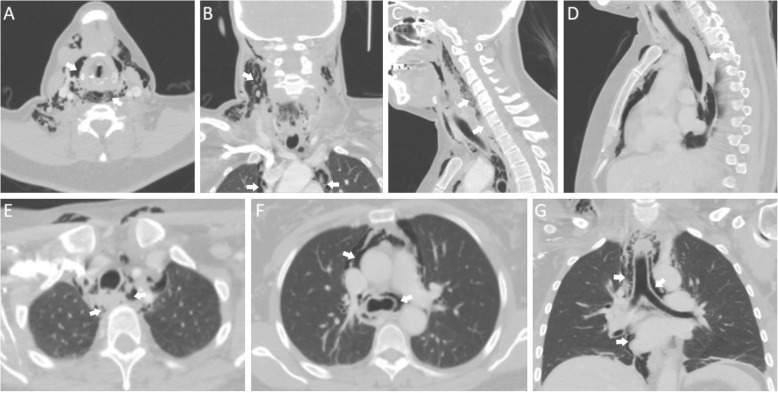
CT scan findings: **a–b**. Extensive emphysema in the soft tissues of the neck. Free air is also visible in the upper mediastinum (white arrows). **c–d**. Sagittal sections showing communication between the esophagus and a false lumen corresponding to the cervical and upper thoracic prevertebral space, containing air and small amounts of fluid (white arrows). **e–g**. Free air in the upper and middle mediastinum, along the intrathoracic esophagus and around the big mediastinal vessels (white arrows)

A 42-year old woman underwent LSG for class 1 obesity (BMI 31 kg/m^2^) in a private hospital in October 2018. Her past medical history was unremarkable, except for psoriasis and a previous laparoscopy for extrauterine pregnancy. The operation lasted 37 min and there were no obvious intraoperative complications. Immediately after the operation the patient started complaining about pain in the throat, mild difficulty swallowing and pain when moving her neck. During the night she complained about chest discomfort. Her vital signs were checked regularly and were normal. The next morning the pain was increasing together with swelling of the throat and marked sialorrhea. These symptoms led to further investigation with laryngoscopy, which was without remarks. A few hours later the patient developed subcutaneous emphysema and subsequently underwent an emergency CT scan, which showed free air outside the esophageal lumen, up to the neck and along the entire intrathoracic esophagus (Fig. 1). The patient was still hemodynamically stable and in good general condition and was transferred to our hospital, which is a tertiary referral center for esophageal surgery, for further management.

**Fig. 2:**
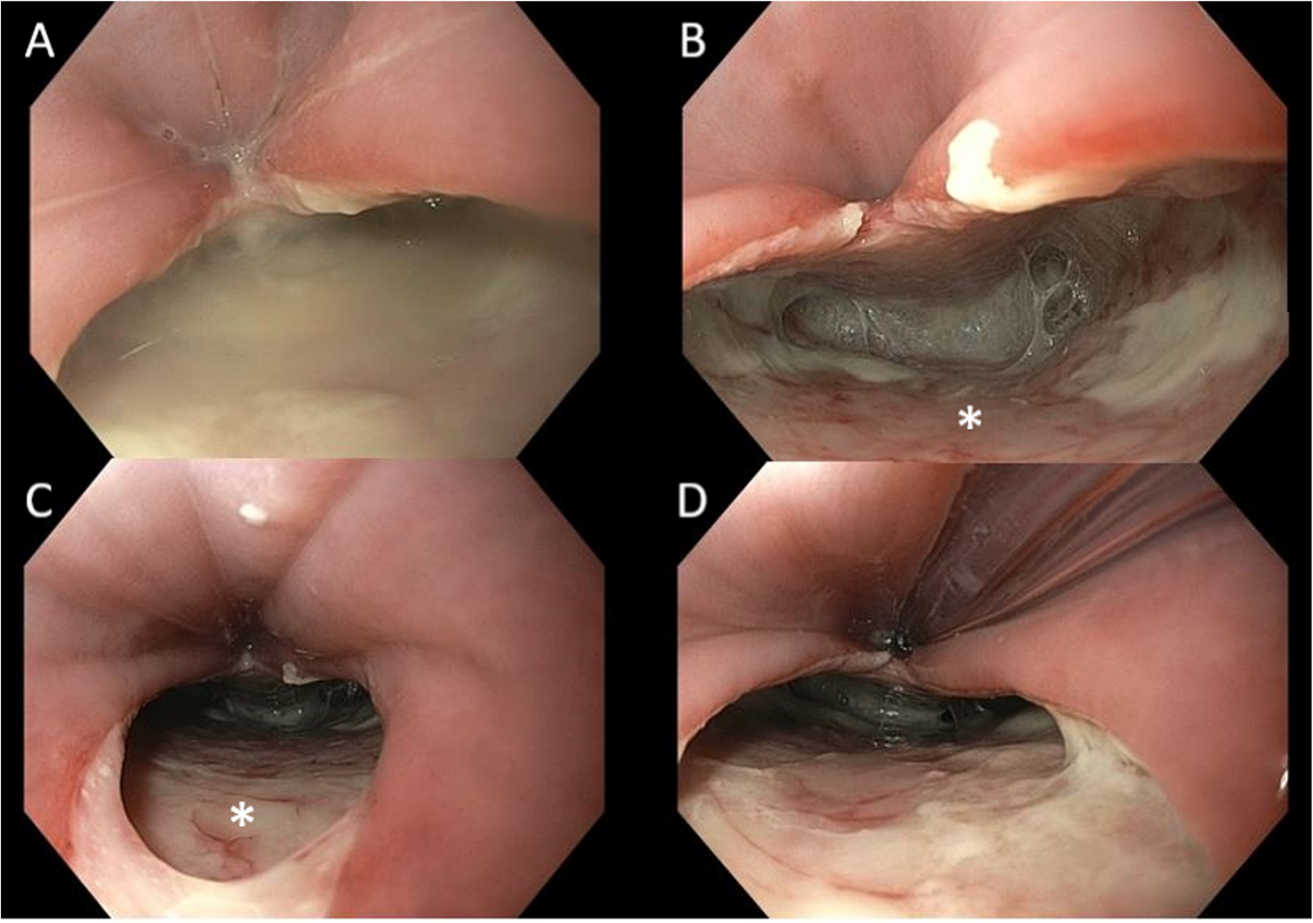
Gastroscopy captures showing a big perforation on the posterior wall of the cervical esophagus. **a**. Large communication with the prevertebral space which is filled with purulent fluid. **b–c**. The prevertebral fascia is visible, marked with an asterisk (*). **d**. A nasogastric tube is inserted under direct vision for decompression

**Fig. 3:**
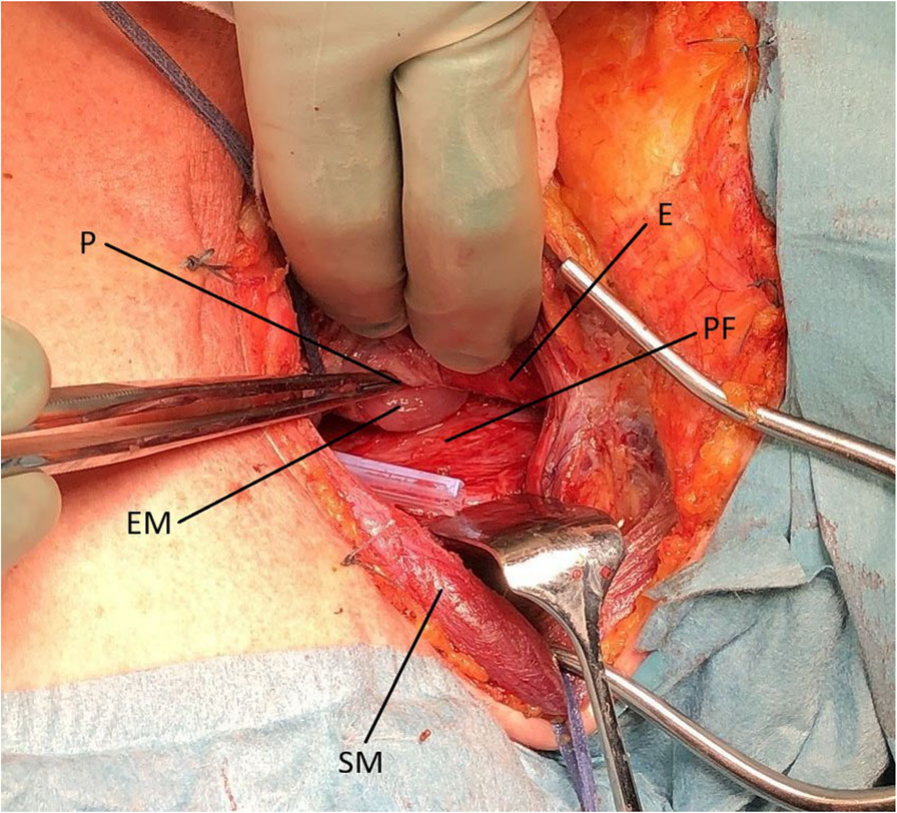
Intraoperative picture showing the perforation on the posterior wall after mobilization of the cervical esophagus. The tip of the thoracic drain that was placed thoracoscopically is visible in the operative field. E: esophagus. P: perforation, with forceps inserted in the defect. EM: esophageal mucosa. PF: prevertebral fascia. SM: sternocleidomastoid muscle

The patient was taken directly to the operating room and a gastroscopy was performed under general anesthesia, revealing a 3 cm long perforation located 14–17 cm from the incisors on the posterior wall of cervical esophagus (Fig. 2). Due to the proximity to the cricopharyngeal muscle, sealing of the perforation by placement of a fully covered esophageal stent was not possible. Additionally, use of Eso-SPONGE® Endoluminal Vacuum Therapy System (B. Braun, Germany) was considered not feasible because of the large size of the defect. Based on the gastroscopy findings, the perforation was estimated to be at exactly the level of the thoracic inlet. Nevertheless, the false lumen was extending more distally, and the prevertebral space was at the time of surgery filled with purulent fluid (Fig. 2). Our assessment was that we needed access to the upper mediastinum to achieve satisfactory drainage and, hopefully, also repair the defect at the same time if possible. The patient was placed in the prone position and a right-sided thoracoscopy was performed. The intrathoracic esophagus was mobilized by incising the mediastinal pleura and a large mediastinal fluid collection was evacuated. The lower border of the perforation high up on the posterior wall of the esophagus at the level of thoracic inlet could be visualized but suturing was technically not possible thoracoscopically. After placing two drains in the thoracic cavity, the patient was placed on the supine position and the cervical esophagus was approached through a left-sided neck incision. The proximal esophagus was mobilized and rotated giving access to the posterior side, the defect was visualized and repaired with interrupted 4/0 PDS sutures (Fig. 3). Further reinforcement was applied by a muscle flap constructed from the sternal head of the sternocleidomastoid muscle. Due to neck edema, delayed extubation was performed as a precaution, and the patient spent the first post-operative day in the Intensive Care Unit but could be discharged and transferred to the ward the following day. Further treatment with nil by mouth, broad-spectrum antibiotics and parenteral nutrition was carried out. The recovery was uneventful; the patient could start an oral diet on post-operative day 6 and was discharged from the hospital 2 days later, on postoperative day 8.

## Literature review

A systematic web-based search using the PubMed, MEDLINE and EMBASE databases was performed, reviewing all medical literature published between 1st January 2000 and 31st July 2019. Keywords and medical subject headings (MeSH) used and combined were: “sleeve gastrectomy”, “esophageal perforation or rupture”, “bougie”. The search was limited to studies published in English. References of the selected articles were checked manually for additional relevant studies. A total of 62 articles were identified, but only two case reports describing iatrogenic perforations caused by the use of a bougie during bariatric surgery were found, both describing more distal lesions located in the middle [4] and lower esophagus [5], respectively. Additionally, a retrospective descriptive study on thoracic complications after bariatric surgery had reported on three patients where thoracic esophageal perforation occurred due to bougie advancement during LSG [6].

## Discussion and conclusions

Bariatric surgery is recognized as the obesity treatment with the most effective long-term results. Among various bariatric procedures, LSG has become the most frequently performed worldwide for several reasons: its simplicity, with a limited alteration of normal anatomy, the excellent weight loss and remission of most obesity-related comorbidities (such as diabetes, hypertension and NASH) and its proven low morbidity compared with some of the other bariatric operations [7–9]. The most common peri-operative complications are staple-line insufficiency (1.3%) and bleeding (1.8%) [10]. Esophageal perforation on the other hand seems to be very rare, since the operation does not directly affect the esophagus.

The standard technique involves the transoral insertion, usually by the anesthesiologist, of a bougie which is advanced to the pylorus and positioned against the lesser curvature, in order to ensure correct size of the gastric tube. A large variety of bougies exist, sizes ranging from 32– 50Fr, with 36Fr being the most commonly used [10]. In our case, a PVC (polyvinyl chloride) gastric tube with a diameter of 35Fr and 80 cm in length was used as a bougie and caused the perforation on the posterior wall of the cervical esophagus. When reviewing the patient’s chart, there was no report on any difficulties positioning the tube and nothing else unusual was noted by the anesthesiology team. A preoperative upper endoscopy was not done and is not routinely performed in patients undergoing bariatric procedures, as current evidence does not support this practice [11]; hence, it is not possible to know if this patient had a preexisting esophageal pathology (e.g. an esophageal diverticulum) present at the time of surgery, which could have predisposed to this complication. This kind of complication is extremely rare, which to some extent explains the delay in diagnosis. However, any deviation from the anticipated postoperative course after bariatric surgery should entail a higher index of suspicion for a possible complication, esophageal perforation being one.

The mortality following esophageal perforation is high, reaching up to 80%, due to the development of mediastinitis and sepsis. Several parameters influence the clinical outcome, including the etiology of the perforation, the size and location of the defect and the time elapsed between the onset of the perforation and the initiation of adequate treatment [12]. These parameters, together with the patient’s comorbidities, general condition and grade of sepsis, also guide the choice of further management. The first step of the workup is usually a CT scan with orally administered contrast, which has a sensitivity of 92–100% for esophageal perforations and can reveal pneumomediastinum, pleural effusion, pneumo- or hydropneumothorax, subcutaneous emphysema and/or pneumoperitoneum. Upper gastrointestinal swallow study with water soluble contrast is used to lesser extent compared to CT scan because of its lower sensitivity, which is around 50% for the detection of cervical perforations and 75–80% for thoracic perforations [13].

The most important principles of the management of an esophageal perforation include resuscitation of the patient, assessment of the defect and timely decision-making regarding operative or non-operative management. If the patient is hemodynamically stable and the mediastinal or abdominal contamination limited, an endoscopy can be performed to assess the perforation and if appropriate treat it with the placement of an esophageal stent [14, 15], endoscopic clips or Eso-SPONGE® in case of small defects [16, 17]. In these patients, a conservative approach can be attempted, consisting of nasogastric decompression, parenteral nutrition, adequate drainage of mediastinal and pleural collections and broad-spectrum antibiotics [18].

In cases where non-surgical management is not feasible, due to the location or size of the perforation or the extent of the contamination, patients should be treated with emergency surgery. The first choice for the surgeon should be the primary suturing of the defect, sometimes with an intercostal or other muscle flap to reinforce the repair. When this is not possible, due to severe sepsis with hemodynamic instability, large size of the defect or friability of the surrounding tissues, the best choice is a damage control approach, e.g. esophageal diversion, which gives the patient the best chance of survival. In these cases, reconstruction of the esophagus is typically performed 6 months to 1 year following the perforation, pending full recovery [19]. In addition to infection source control, adequate external drainage of all mediastinal and thoracic collections is mandatory.

In our case, the patient was stable but endoscopic treatment of the perforation was not deemed feasible; the defect was large but surrounded by vital tissue, so we decided to perform thoracoscopy in order to wash out the thoracic cavity and repair the defect. Nevertheless, the perforation was located too high to be repaired thoracoscopically and a neck incision was necessary in order to gain access. We performed a primary closure of the defect with sternocleidomastoid muscle flap reinforcement, with drainage of the mediastinum and thoracic cavity and broad-spectrum antibiotic therapy. The post-operative course was uneventful with a successful outcome.

In conclusion, this is the first reported case, to our knowledge, of a cervical esophageal perforation caused by the bougie during LSG. We present it in order to underline that the risks inherited in the use of a bougie (or any other esophageal tube) during surgery should not be underestimated and that the insertion must be done with extreme caution. Esophageal perforation is still a challenging, life threatening, complication. Once the suspicion of a perforation arises all available tests (CT scan with orally administered water-soluble contrast and gastroscopy) should be performed without delay, as prompt diagnosis and initiation of adequate treatment is the key to a favorable outcome.

## Data Availability

Not applicable.

## Abbreviations

BMI: Body mass index
CT: Computed tomography
LSG: Laparoscopic sleeve gastrectomy
NASH: Nonalcoholic steatohepatitis
PVC: Polyvinyl chloride
